# Comparative quality analysis and economic feasibility of solar assisted yogurt processing unit for decentralized dairy value chain

**DOI:** 10.1038/s41598-023-34032-y

**Published:** 2023-04-27

**Authors:** Syed Nabeel Husnain, Anjum Munir, Waseem Amjad, Faizan Majeed, Oliver Hensel

**Affiliations:** 1 Department of Agricultural and Biosystems Engineering, University of Kassel, 37213 Witzenhausen, Germany; 2 Department of Energy Systems Engineering, University of Agriculture, Faisalabad, 38000 Pakistan; 3 Department of Agricultural Engineering, Bahauddin Zakariya University, Multan, 60800 Pakistan

**Keywords:** Energy science and technology, Engineering

## Abstract

Due to the lack of farm-gate milk processing facilities, dairy farmers have to sell raw milk, resulting in economic and quality compromises. The study compared the quality of yogurt processed in solar assisted yogurt processing unit with the existing milk value chain and its techno-economic feasibility. For this, an investigation of the experiment was executed where four different milk processing approaches were compared. The quality attributes for processed milk like fat (5.283%), solid-not-fat (9.0833%), salts (0.6833%), protein (3.8%), lactose (4.1%), total solids (14.383%), pH (6.87), density (1.031 kg/L) and freezing point (− 0.532 °C) were found within the standardized ranges. Similarly, for the case of yogurt, these attributes were found as fat (5.5%), solid-not-fat (8.683%), acidity (0.93%), lactose (4.73%), total solids (14.183%), pH (4.3433), density (1.039 kg/L) syneresis (9.87 mL/100 g), *S. thermophilus* count range (10.18–10.30 log cfu/mL) and *L. bulgaricus* count range (10.26–10.34 log cfu/mL). Moreover, no detection of coliform count in solar-processed yogurt, endorsed the current idea to perform three processes of heating, fermentation, and cooling in a single unit. Based on the energy sources utilized, the payback period was calculated to be 1.3–9 years with an expected lifespan of 15 years while in terms of product profit, the payback period was predicted to be 1.78 years. The processing cost per liter of milk for yogurt production was calculated to be 0.0189 USD. Considering CO_2_ emission savings, it is anticipated that a solar-powered yogurt processing unit can generate 107.73 MWh of useful energy during its operating life with zero CO_2_ emission.

## Introduction

Yogurt is one of the oldest fermented milk products, and it is widely consumed around the world. It contains a lot of protein, calcium, and vitamins. Lactic acid-producing bacteria, such as *S. thermophilus* and *L. bulgaricus*, or other bacteria with mutually complementary metabolism, ferment yogurt^1^. Natural yogurt has a delicate walnutty flavor and a smooth and viscous gel-like texture^2^. Lactic acid bacteria ferment lactose, producing lactic acid, carbon dioxide, acetic acid, diacetyl, acetaldehyde, and a variety of other compounds that give yogurt its distinct flavor^3^. Hamdan et al.^4^ found that a 1:1 mixture of *L. bulgaricus* and *S. Thermophilus produced high* acetaldehyde in milk. However, producing safe and high-quality yogurt necessitates meticulous processing. In reality, even a small amount of contamination can degrade the quality of the yogurt and have serious health consequences for consumers.

Pakistan produces over 59.666 million tonnes of milk annually ranking third in the world after India and the United States^5^, with the bulk of producers being small-scale farmers (> 80%). Unfortunately, only 5% of this milk is processed, with the rest being handled by milkmen who are frequently unsanitary and pose significant health concerns. Due to a lack of processing facilities, 15–19% of the total milk produced in the country is wasted, while the rest is handled incorrectly^6^. Not only in the Indo-Pak subcontinent, yogurt is a popular dairy product but also worldwide i.e. yogurt production increased by 8.3 × 10^6^ tonnes during the period from 1990 to 2015 in the United States^7^. In Pakistan, yogurt accounts for over 70% of all fermented dairy products^8^, although milk fermentation receives less attention in order to improve shelf life, aroma, and nutritional content. Table 1 enlist the top milk^5^ and yogurt^9^ producer countries.

**Table 1:** Top milk and yogurt producing countries in the world.

Milk production in 2021		Yogurt production in 2020	
Country	Million metric tonnes	Country	Million metric tonnes
India	208.984429	United States	9.91
United States	102.654613	Turkey	5.92
Pakistan	59.666000	India	5.76
China	41.707232	Brazil	5.31
Brazil	36.663708	Germany	5.17
Russia	32.333278	France	3.9
France	25.834800	Iran	2.96
Turkey	23.200306	Russia	2.86
New Zealand	21.886376	United Kingdom	2.57
United Kingdom	15.221000	Italy	2.28

Unfortunately, several chemical and microbiological adulterants degrade milk quality throughout processing and along the supply chain^10–14^. In developing countries, milk production and distribution systems are still very traditional and dominated primarily by the informal private sector, which consists of various agents such as producers, collectors, middlemen, processors, traders, and dairy shops, each of whom performs a specialized role at a specific point in the supply chain^15^. Practically, at every stage of the marketing process, there is almost no testing^16^. The majority of milk businesses in urban areas are exposed to dust and insects, and just a handful of them are equipped with refrigeration. The transportation containers are unsanitary, and milk adulteration is a major concern in the peri-urban milk supply chain. However, due to price-conscious customers in the country, demand for raw milk and its products, such as yogurt, is higher than for processed milk and its products^17^. Because of the widespread consumption of milk and dairy products, these commodities are possible targets for adulteration, with financial gain for unscrupulous producers^18^.

In Pakistan, a very less amount of processed yogurt (branded) is accessible, and yogurt is primarily produced on a small scale (unbranded) by local people (Gawalas) and is known locally as dahi. Unbranded (dahi) yogurt is made under less controlled conditions than branded yogurt (milk standardization, culture concentration, viability, incubation temperature and time, etc.). Furthermore, there are no clear guidelines for fermented dairy products. As a result, the quality of yogurt/dahi in the local market varies greatly from store to shop. However, people are becoming increasingly aware of the importance of food quality^19^. Many factors influence the quality of processed yogurt. One of the most essential variables is to maintain the proper temperature profile i.e., heating the milk to 80 °C, keeping the temperature of inoculated milk between 40 and 45 °C during fermentation, and then fast cooling of yogurt to below 8 °C^20^. Grigorov^21^ also recommended the pasteurization of milk at 85 °C for 20 to 30 min to minimize syneresis in the yogurt instead of 90–95 °C which causes product deterioration with similar holding times. Rowland^22^ examined how much albumin and globulin denatured when milk was heated at temperatures ranging from 63 to 80 °C for varying lengths of time, and found that 83.4% of the total albumin and globulin denatured after 30 min at 80 °C.

Microbial contamination (pathogens) may occur as a result of unsanitary operating conditions, posing a major health risk to consumers. Customer demand for taste, quality, stability, and shelf life of milk and yogurt, on the other hand, is increasing. As a result, basic research in the field of quality assessment of marketed milk/yogurt is required to raise public awareness. For this purpose, various branded (industrial) and unbranded (locally produced) samples of milk/yogurt were obtained and their quality was analyzed in Faisalabad, Pakistan's third largest city.

Secondly, lack of on-farm processing facilities, dairy farmers are forced to sell high-quality perishable raw milk to local milkmen and large milk collectors at lower prices^23^. In Pakistan, almost 95% of milk is sold in raw form through informal marketing channels, offering the potential for adulteration at every step supply chain^24^. Traditional quality criteria, such as smelling or boiling the milk to identify any curdling or adulteration, are frequently used by processors. Processing is frequently performed in unsanitary conditions. Manual labor, premises rent and fuel, which might range from burning wood to electricity, are all included in production costs. For example, a farm cooling tank with a capacity of 200 L costs USD 3313 and one with a capacity of 1000 L costs USD 6812. As a result, milk is traditionally stored in non-food-grade containers with ice (which may be contaminated) as a refrigerant to prevent spoilage, especially during the summer season^16^. So, the dairy producers' ability to install chilling units and pasteurizers for on-farm dairy processing is hampered by high procurement and operational expenses.

The use of fossil-fuel energy inputs for continuous operation accounts for a considerable share of costs on dairy farms with milk processing facilities. The dairy industry produces around 4% of all anthropogenic greenhouse gases (GHGs), or about 1.2 billion tons of CO_2_ each year^25^. The widespread use of fossil fuels as a primary energy source in dairy processing contributes to pollution, necessitating immediate action to transition dairy processing to renewable energy sources^26^. Pakistan receives a lot of solar energy, 19 MJm^−2^ for 7.6 h a day, with a DNI of 5 to 7 kWh m^−2^ d^−1^ on average^27^. More than a billion people (56 percent) in Pakistan live in rural and remote areas, relying on wood, charcoal, dung cakes, agricultural residue, or carbon-based fuels to meet their energy demands. While over 0.51 billion (27%) are still not linked to the national electrical grid, those that are connected have transmission lines limited to inhabited regions for residential usage exclusively, with the majority of dairy farm operations taking place outside of villages^28^. In a nutshell, developing self-sufficient, viable, and off-grid energy solutions for rural areas, is the need of the time. Therefore, a solar assisted yogurt processing unit has been developed for the decentralized processing of raw milk^20,29^. Although, the ultimate end product is yogurt, but the developed system process the yogurt from raw milk i.e., capable of heating the raw milk which is the prerequisite for yogurt fermentation. Because the quality of the raw milk affects the quality of yogurt and adulteration in the raw milk is expected during its transportation (conventional practice). Therefore, in the current study quality analysis of yogurt as well as raw milk had been conducted for comparison with locally available milk being used for yogurt making at home and local shops. Moreover, the economic feasibility of the system has also been conducted for the adaption in the rural community. It is expected that the technology able to process a quality product with minimum operational expenses, would not only help in reducing post-milking losses but also generate income for end users.

## Methodology

In collaboration with the International Center for Development and Decent Work (ICDD, University of Kassel, Germany and Dairy Industries, Okara-Pakistan, the Department of Energy Systems Engineering, University of Agriculture Faisalabad (UAF) Pakistan has developed and fabricated a complete yogurt processing unit.

### System description

**Figure 1:**
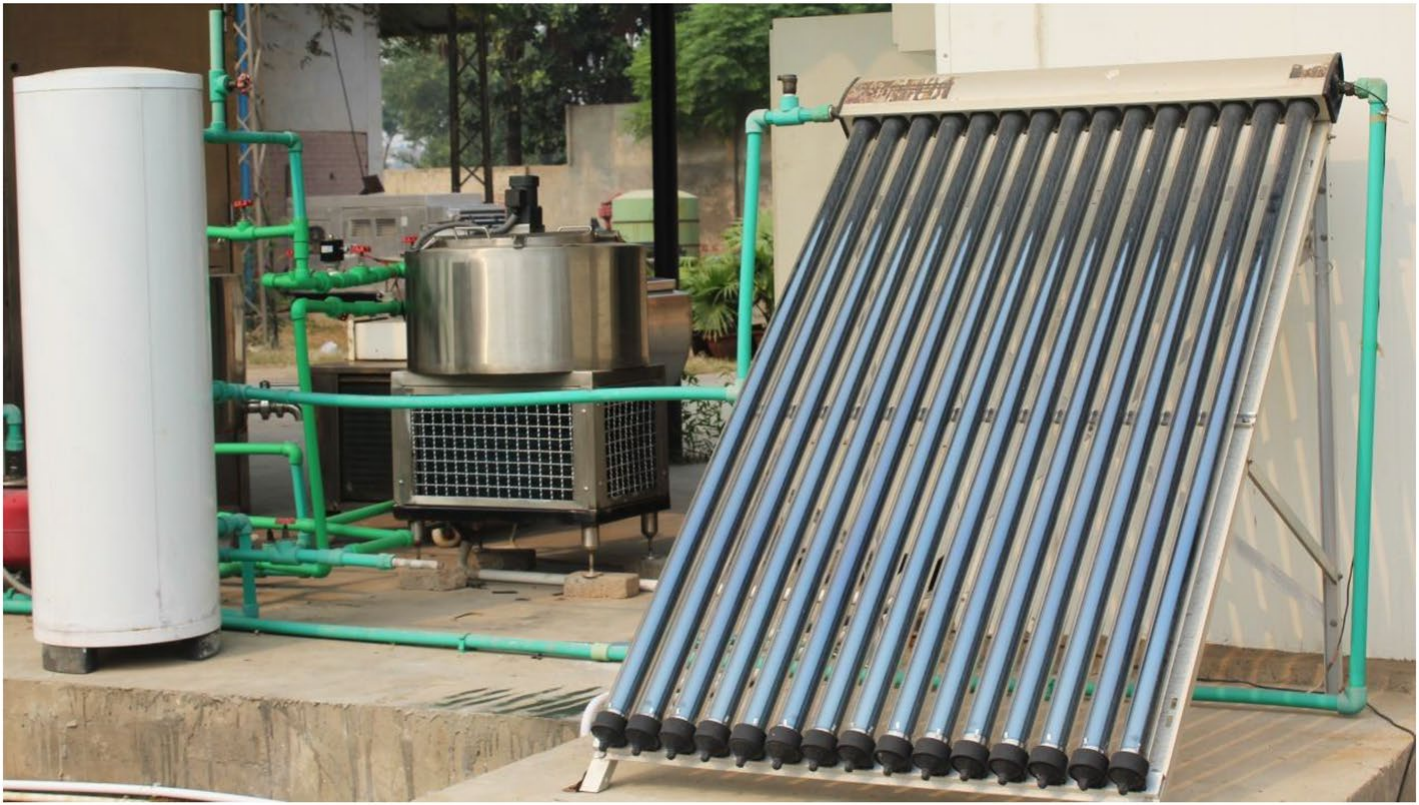
Solar assisted yogurt processing unit^20^.

The design of the yogurt processing unit and the selection of its energy source is mostly dependent on some fundamental factors such as maintenance, energy efficiency, and, in particular, product life cycle and environmental impact. Figure 1 shows a solar-based yogurt processing unit designed to process the raw milk and ferments it into yogurt at the production site in a timely and controlled manner. It consists of a cylindrical fermentation chamber (560 mm diameter and 230 mm depth) with a 50-L capacity made of stainless steel (food grade SS 304) and is surrounded by a heating coil (3.5 m long, 40 mm wide and 12.5 mm high). A pillow plate is placed at the bottom surface of the chamber that works as an evaporator for cooling reasons. Further technical details can be found in Husnain et al.^20^.

**Figure 2:**
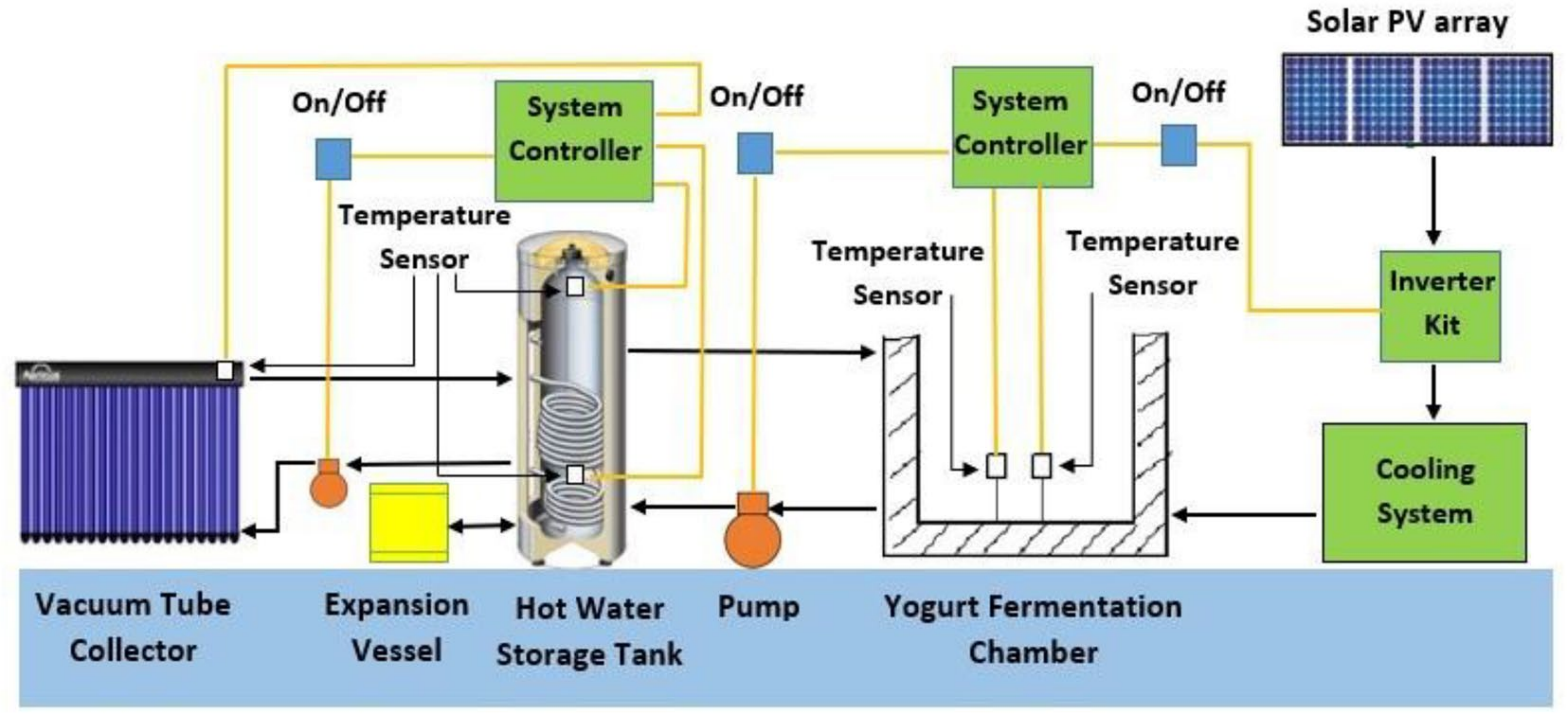
Experimental setup layout of solar assisted yogurt processing unit^20^.

Briefly, to explain the operating procedure, Fig. 2 shows the schematic of the developed unit to elaborate on the components' connectivity. The yogurt processing unit was coupled with a 100 L capacity hot water storage tank which receives heat from a solar evacuated tube collector (2.46 m^2^). A centrifugal pump (Wilo-SP106) was installed for the circulation of propylene glycol solution (50% by volume) between the hot water storage tank and the evacuated tube collector. The pump can operate at three variable speeds (600 L/h, 900 L/h and 1100 L/h) and requires 80 W of power at maximum speed. Another stainless steel, centrifugal water circulation pump (WB50/025D, 50 L/min.) was installed between the outlet of the hot water storage tank and the inlet of the yogurt processing unit to circulate the hot water through the square spiral coil heat exchanger to increase milk temperature up to 80 °C. Because the system is closed, an expansion vessel (12 L) was included to prevent high-pressure build-up. When the temperature differential between the water–glycol solution leaving the evacuated tube collector and the water in the lower portion of the hot water storage tank exceeds 5 °C, the controller turns on the circulation pump (Wilo-SP106) and turns it off when the differential is below 5 °C or when the water temperature in the storage tank exceeds 90 °C.

### Experimental layout and data collection

The study was carried out in the region of Faisalabad, Pakistan, to assess the quality of local shop yogurt, homemade yogurt, branded/company-processed yogurt, and solar-processed yogurt in order to make a thorough quality comparison. The experimental protocols included the heating of the three different quantities of raw milk (50, 40 and 30 L) up to 80 °C at the continuous stirrer speed of 36 rpm which took about 140–80 min depending upon the quantity to be processed. After that, tap water was passed through the heating coil in an open loop under the supervision of manually operated valves to lower the temperature of the heated milk to 43 °C, which is recommended for milk fermentation. The starter culture was inoculated (2–3% of the volume of milk) at this temperature, and the temperature was maintained by a solenoid valve controlling the hot water circulation for 5–6 h until the requisite pH (4.85–4.5) was reached. After that process, the refrigeration system was turned on to bring down the temperature of yogurt below 8 °C which is essential to increase the shelf life by reducing the bacterial activities which normally took 48–103 min depending upon the quantity of the processed milk and the stirrer speeds (36, 18 and 6 rpm). Temperatures at the intake and exit of the evacuated tube collector, the top and bottom of the hot water storage tank, and inside the fermentation chamber were measured using a controller with Resistance Temperature Detector (RTD) based temperature sensors. As the milk was fermenting, the pH was measured using a portable pH meter (ML1010). A clamp meter (Fluke 345PQ) and pyranometer (METEON) were used to access the performance of the installed PV system. Furthermore, CIP was carried out following each experiment. A detailed description of this process is reported by Husnain et al.^20^.

**Figure 3:**
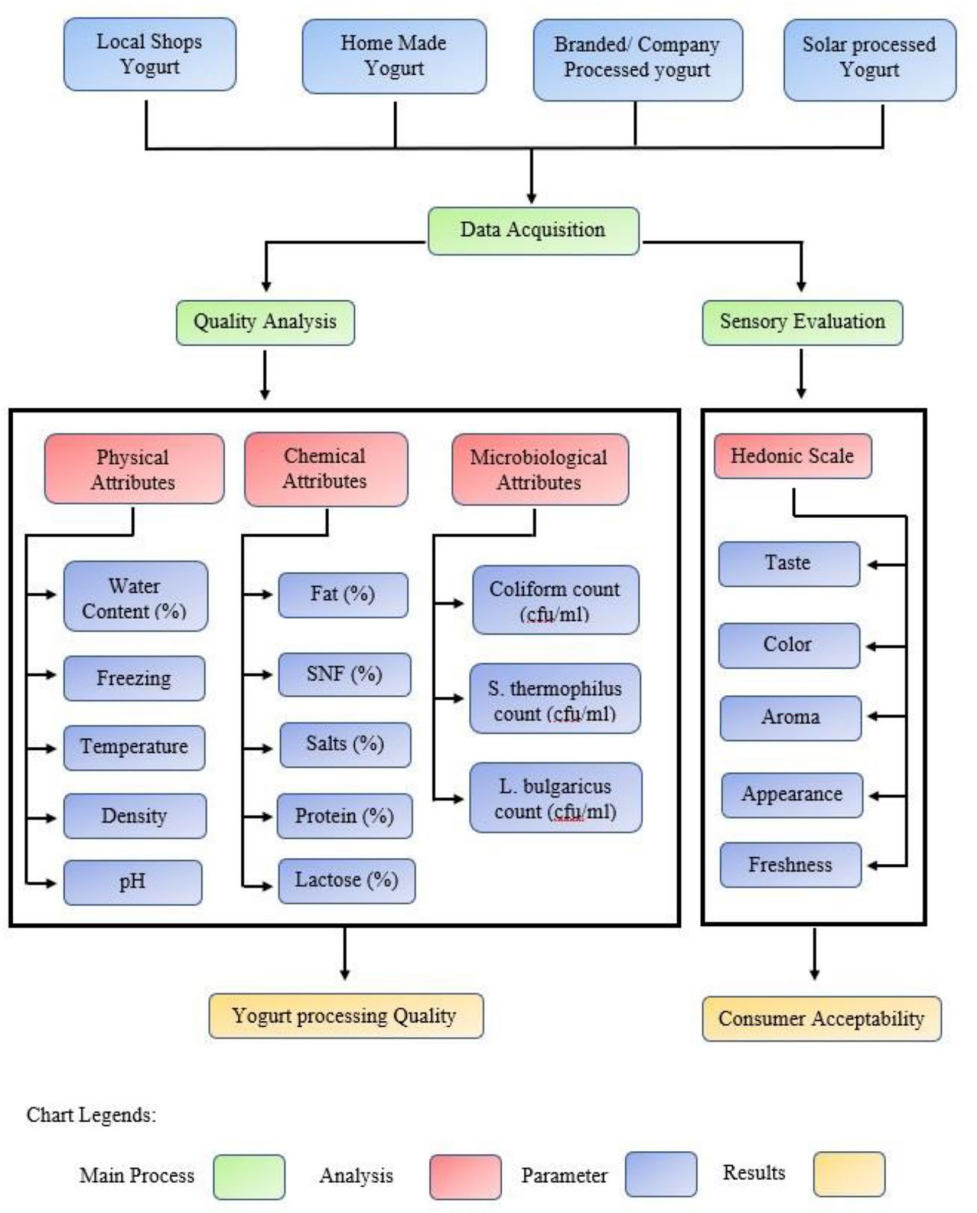
Flowchart of research methodology.

The study used an ultrasonic milk analyzer (Master Pro P1, Milkotester Ltd.) to determine physical attributes such as water added (W, %), freezing point (Fp, °C), temperature (T, °C), density (ρ, kg/L) and pH, and chemical attributes: fat (Ft., %), protein (Prot., %), salts (Sal., %), solids-not-fat (SNF%) and lactose (Lac., %) with a testing capacity of 50 samples per hour. A milk analyzer was used to examine the milk quality of randomly selected open milk/yogurt-selling shops (20), milk for homemade yogurt (20), and branded/company-processed milk (10). Under sterilized conditions, three samples of milk and already fermented yogurt were collected at the same time from each selected local shop and milkmen in Faisalabad. Each branded milk and yogurt sample yielded three random samples. Unbranded samples were gathered in sanitized vials, whereas branded samples were kept in their original packaging. The samples were tested as soon as possible after they were collected. The quality of yogurt processed with a newly developed solar yogurt processing unit was then compared to the data collected. All of the data was collected in duplicate. Figure 3 depicts a flowchart of the research technique. The economic viability of the solar yogurt processing unit was studied using the straight-line approach and per liter milk processing cost after quality analysis. Furthermore, the reduction in carbon emissions over the lifetime of the newly developed machine has been calculated.

### Quality analysis

An ultrasonic milk analyzer was utilized to check the quality of the milk used for yogurt fermentation (Master Pro P1, Milkotester Ltd.). The milk analyzer was calibrated for the local herd according to the standard protocols at the National Institute of Food Science & Technology (NIFSAT), University of Agriculture Faisalabad (UAF), Pakistan, to ensure the correctness and reliability of the results^30^. Although numerous physio-chemical parameters such as W, Fp, T, ρ, pH, Ft, Prot., Sal., SNF, and Lac. were examined with an ultrasonic milk analyzer, lab testing was also done for calibration purposes. The fat content of milk/yogurt was determined using the Gerber method, as described by Pearson^31^, and the protein content was determined using the Kjeldahl method, as reported by the AOAC^32^. The total solids (TS, %) were determined using the AOAC^32^ and to determine the SNF content present in a given milk sample Harding^33^ technique was used. The lactose content of the milk sample was investigated by the following formula^24^:

1 $$Lactose\,\left( \% \right) = TS\,\left( \% \right) - \left( {Ft\% + Prot.\% + Ash\% } \right)$$

According to the AOAC^32^, the ash content (%) was determined using a gravimetric method in a Muffle furnace at 550 °C. The Methylene Blue Reduction Test was used to determine the sanitary state of milk/yogurt. Standard techniques were used to detect various milk/yogurt adulterants such as water, starch, urea, formalin, hydrogen peroxide, detergents, oil, and cane sugar^34^.

#### Synersis by centrifugal method

The procedure described by Hassan et al.^35^ was used to separate whey from yogurt samples. At 5 °C, 25 mL of set yogurt was progressively transferred to 50 mL centrifuge tubes, causing minimal coagulum disruption. The centrifuge tubes were weighted and centrifuged for 20 min at 3394 RPM in an eppendorf (5804 R) centrifuge (manufactured in Germany). In centrifuge tubes, the amount of whey separated at the top of the coagulum was measured in milliliters. Whey syneresis was measured using the weight fraction of the supernatant liquid (mL/100 g yogurt). The whey separation was proportional to the volume of the whey separated, and vice versa.

#### Microbiological analysis

The total viable count of *S. thermophilus, L. bulgaricus*, and Coliform was determined using the Coppuccino and Sherman^36^ standard plate count method. According to Harrigan and McCance^37^, the selective media utilized for a viable count of *S. thermophilus, L. bulgaricus*, and Coliform were Neutral red chalk lactose agar, Acetate agar, and Violate red bile agar, respectively.

### Statistical analysis

All the data were taken in triplicate and Fisher's analysis of variance was used to statistically examine the data obtained on various parameters using the computer program MINITAB (2018). The differences between the means of the treatments were compared using the least significant difference (LSD) test with 0.05 probability levels. (Steel et al. 1997)^38^.

### Economic analysis

Economic viability, in addition to technical soundness, is a significant factor in the successful adoption of developed technology by milk producers and processors. As a result, using the straight-line method, yogurt processing unit^20^ has been economically evaluated based on the payback period and revenue generated during the usable lifespan^39^. Fixed costs included the initial investment, depreciation (Eq. 2), interest (Eq. 3), insurance (2%), taxes (1.5%), and housing costs, as well as operational expenditures such as labor, operating (10%), and repair and maintenance charges (25%). Housing and labor costs were not included since it was assumed that the milk producers would process the milk utilizing developed technologies at the farm level.

2 $$Depriciation = \frac{Initial\;cost - Salvage\;value}{{Years\;of\;useful\;life}}$$

The salvage value was set at 10% of the original investment, and the projected life was set at 15 years. The following equation used to compute interest using the annual interest rate in Pakistan (7%) as a factor.

3 $$Interest = \frac{{\left( {Initial\;cost + salvage\;value} \right) \times annual\;intrest\;rate}}{2}$$

Payback periods were calculated using a break-even approach. The break-even point, according to Munir et al.^40^, is the time it takes to equalize total cost (fixed and operational) and revenue (in terms of cumulative fuel savings and product profit), after which the machine begins to generate income in terms of fuel savings. Because weather conditions affect daily useable working hours, all economic estimates were done on an hourly basis.

### Carbon emission analysis

Because the developed yogurt processing unit^20,29^ is totally solar-powered, there has been no carbon emission. In comparison to fossil-fuel-based energy generating resources, a carbon saving study was also conducted to estimate CO_2_ emissions. For this reason, the total energy used by developed technologies throughout their operational hours across their lifetimes was calculated, and CO_2_ emissions for non-renewable energy resources were computed if they were employed to create the same amount of energy. Quaschning^41^ published the CO_2_ emissions per kWh of energy generation using various fossil fuels, which were used to calculate the CO_2_ emissions generated by these fuels for equivalent energy generation.

## Results and discussion

### Quality analysis

#### Physical attributes

##### pH

The mean pH value of milk samples collected from sources other than solar-processed milk ranged from 6.53 to 6.60 (Table 2) and was found to be within normal limits. Several researchers reported similar findings^42,43^. The addition of ice, water, or any other chemical preservative to extend the perishability of pure raw milk could be the cause of lower pH values in market milk samples^24^. Solar-processed milk (6.87 + 0.0404) had the greatest pH and was closest to the mandated pH since it was pure and fresh with no impurities. The pH of all branded and solar-processed yogurt samples, on the other hand, was over 4, whereas the pH of local shops and handmade yogurt samples was even lower than 4, resulting in increased acidity (Table 3). In fact, unchecked fermentation results in a lower pH and increased acidity. Furthermore, unbranded yogurt lacks an appropriate culture dosage mechanism, which has a significant impact on the acidity of the finished product^44^. Solar-processed yogurt (4.3433 + 0.0521) had the greatest pH and was the closest to the mandated pH since it was pure and fresh with no pollutants.

**Table 2:** Physical attributes of milk samples tested from local shops, milkmen, company-processed milk and solar-processed milk.

Milk source		pH of milk	Density (kg/L)	Freezing point of milk (°C)	Fat (%)	Total solid (%)	Solid-not-Fat (%)	Salts %	Protein %	Lactose %
		Mean ± SE (%)								
Local market available milk	Local shops	6.5300 ± 0.589	1.0280 ± 0.00520	− 0.4490 ± 0.00404	1.7830 ± 0.344	7.4830 ± 0.188	5.6830 ± 0.407	0.4833 ± 0.0837	2.1	2.4
	Milkmen supplied milk	6.6000 ± 0.421	1.0290 ± 0.00231	− 0.4630 ± 0.00115	2.6830 ± 0.551	8.28670 ± 0.0924	5.5830 ± 0.159	0.3833 ± 0.0433	2.2	2.6
Company processed milk		6.710 ± 0.0808	1.0310 ± 0.000577	− 0.5180 ± 0.00115	3.4830 ± 0.193	12.1830 ± 0.118	8.6830 ± 0.101	0.6833 ± 0.0549	3.2	4
Solar processed milk		6.8700 ± 0.0404	1.0310 ± 0.000577	− 0.5320 ± 0.000577	5.2830 ± 0.130	14.3830 ± 0.0606	9.0833 ± 0.0722	0.6833 ± 0.0318	3.8	4.1

**Table 3:** Physical attributes of yogurt samples tested from local shops, milkmen, company-processed milk and solar-processed milk.

Yogurt source		Density (kg/L)	pH of Yogurt	Acidity (%)	Lactose (%)	Fat (%)	Total Solids (%)	Solid-not-Fat (%)	Syneresis (mL/100 g)
		Mean ± SE (%)							
Local market available yogurt	Local shops	1.0340 ± 0.00173	3.9100 ± 0.242	1.4670 ± 0.0751	4.3300 ± 0.139	1.8833 ± 0.0953	7.4830 ± 0.193	5.5830 ± 0.263	13.2200 ± 0.323
	Yogurt fermented by milkmen supplied milk at homes	1.0360 ± 0.00115	3.9600 ± 0.139	1.1067 ± 0.0578	4.3500 ± 0.121	2.5470 ± 0.262	8.1830 ± 0.130	5.2830 ± 0.188	12.1100 ± 0.294
Company processed yogurt		1.0400 ± 0.00115	4.1933 ± 0.0780	0.9600 ± 0.0462	4.6400 ± 0.0751	3.4800 ± 0.0693	12.2830 ± 0.118	8.7830 ± 0.170	9.3500 ± 0.133
Solar processed Milk and yogurt		1.0390 ± 0.000577	4.3433 ± 0.0521	0.9300 ± 0.0346	4.7300 ± 0.0404	5.5000 ± 0.0462	14.1830 ± 0.0722	8.6830 ± 0.107	9.8700 ± 0.0520

##### Freezing point (Fp)

Locally marketed milk had the widest freezing point range, ranging from − 0.449 ± 0.00404 to − 0.463 ± 0.00115, as shown in Table 2, followed by company treated milk for yogurt fermentation (− 0.518 ± 0.00115) and solar processed milk (− 0.532 ± 0.000577). Individuality, breed variances, acquired acidity, colostrum, mastitis, lactation stage, nutrition, and season can all impact the freezing point of milk^45^. Also, the presence of mixed water in the local market milk can be linked to the samples' greater freezing point, as the current investigation found that the local market milk contained a higher percentage of added water.

##### Density (ρ)

The lowest density range has been reported in local market available milk and yogurt (1.028 ± 0.0052 to 1.029 ± 0.00231) and (1.034 ± 0.00173 to 1.036 ± 0.000577) respectively, presumably due to the dilution of water in raw milk^24^. Company-processed milk and yogurt (1.031 ± 0.000577) and (1.04 + 0.00115), as well as solar-processed milk and yogurt (1.031 ± 0.000577) and (1.039 ± 0.000577) respectively as shown in Tables 2 and 3, were determined to be more consistent with the Pakistan Pure Food Rule 1965's specified density range of milk^46^. The inclusion of binding agents and preservatives for a longer shelf life may be the cause for the higher density of company-processed yogurt.

##### Temperature (T)

The samples' temperatures during testing for local milk stores, milkmen, and company-processed milk ranged from 28.9 to 30.3 °C, with an average sample temperature of 29.2 °C, and were confirmed to be within the milk analyzer's testing conditions^30^.

#### Chemical attributes

On the basis of different chemical properties, the results of solar yogurt processing processed milk and yogurt are compared with available in local market and corporate processed milk and yogurt samples and are illustrated in Tables 2 and 3, respectively.

##### Fat (Ft)

As shown in Tables 2 and 3, the fat percentage of locally available milk and yogurt ranged from 1.783 to 2.683% and 1.8833 to 2.547% respectively and had the lowest values of all the other examined sources, indicating that cow milk and yogurt had the lowest fat percentage. These decreased fat % findings could be due to suspected adulteration of cow milk with water. In previous research, the same adulteration causes were described^24,47^. Skimming or partial skimming of milk is a frequent practice in local milk processing plants, resulting in lower fat content in milk and its derivatives. Variable fat percent can also be caused by differences in breed, type and quality of feed, environmental factors, and genetic variability^33,47^. Solar-processed milk and yogurt, on the other hand, had the highest fat percentages of 5.3 and 5.5%, which is similar to the industry standard, and were followed by corporate processed milk and yogurt (3.5%)^46^.

##### Solid-not-Fat (SNF)

The SNF (%) of corporate processed and solar processed milk/yogurt was determined to be 8.68% and 9.0833% for milk and 8.783% and 8.683% for yogurt, respectively, which falls within the Pakistan Pure Food Rule 1965's recommended standard^46^. The inclusion of preservatives and binding agents for longer shelf life and thicker yogurt production may account for the greater SNF value in company-processed yogurt. According to Awan^46^, the results for local market milk and yogurt SNF (%) did not meet the legal minimum requirement (Tables 2, 3), but were significantly lower than cow milk (8.50%). These findings are congruent with the findings of a recent study, which indicated that local market milk samples were consistently contaminated with water or cow milk since they had greater freezing points.

##### Salts (Sal.)

Phospholipids, chlorides, carbonates and bicarbonates of sodium, potassium, calcium, and magnesium, among other salts, are found in milk. A milk analyzer was used to determine the overall concentration of salts in the milk samples, and the results revealed that salts were identified in all of the examined samples in the range of 0.3833 to 0.6833 percent. As shown in Table 2, lower salt percentages were found in local shops and milkmen's sold milk, whereas the highest salt percentages were found in business and solar-processed milk. The findings were found to be comparable to those of Abd El-Salam and El-Shibiny^48^.

##### Protein (Prot.)

Solar-processed milk (3.8%) had the highest protein content (%), followed by company-processed milk (3.2%) and local market-processed milk (2.1–2.2%), as shown in Table 2. The protein level of solar and market-processed milk was found to be in compliance with quality criteria^46^. However, differences in protein content (%) can be ascribed to processing quality and management approaches.

##### Lactose (Lac.)

As shown in Table 2, lactose levels were highest (4.1%) in milk utilized in solar yogurt processing units for yogurt production and lowest (2.4–2.6%) in local market milk. Lactose content was determined to be 4.0% in company-processed milk. Sharif, et al.^49^ linked the severity of sub-clinical mastitis to a fall in lactose (%) in Pakistani buffalo milk, but the most relevant rationale for the current study could be milk adulteration, resulting in lower lactose levels in local market milk.

##### Total solids (TS).

Local shops and milkmen supplied milk and yogurt had total solids contents of 7.483 ± 0.188% and 8.2867 ± 0.0924% for milk, and 7.483 ± 0.193% and 8.183 ± 0.130% for yogurt, respectively, which was much lower than the average value of company processed milk and yogurt (12.2 ± 0.24% and 12.3 ± 0.24%) and solar processed milk and yogurt (14.383 ± 0.0606% and 14.183 ± 0.0722%) and did not meet the quality standards^46^. As shown in Tables 2 and 3, the solar-processed milk and yogurt had the highest standardized TS (%), followed by the company-processed milk and yogurt.

#### Microbiological analysis of yogurt

The microbiological quality assessment of yogurt is primarily concerned with two aspects: (1) consumer protection from health hazards, and (2) ensuring that the material does not suffer microbial deterioration throughout its expected shelf life^50^. In reality, it aids in determining the extent to which hygienic precautions were taken during processing, allowing for the forecast of product shelf life and the detection of potential health risks (pathogens).

The presence of coliforms (6–15 cfu/mL) in branded yogurt samples was discovered by microbiological examination, indicating some type of mistreatment (should be ≤ 10 cfu/mL or 1 log cfu/mL) even inside the industry. In local shop yogurt samples, however, a larger number (1.93–2 log cfu/mL) and (1.34–1.43 log cfu/mL) of coliforms were found, indicating a high level of mishandling. The greater coliform level may be due to the filthy conditions that existed during the production process. Furthermore, this figure may include contamination from the post-processing stage. On the other hand, yogurt produced by a solar-assisted yogurt processing unit had no traces of coliform, indicating a high level of hygienic processing conditions. This supports the current study's idea of performing all processes (heating, fermentation, and cooling) in a single container to reduce the risk of contamination. The system is compact named as 3 in 1 capable to perform all the required processes (Heating, Fermentation and Cooling) in a single container. So, there is less chance of contamination during the transfer of heated milk into the fermentation container, which is conventional practice. Moreover, the chamber is completely closed to avoid foreign contamination. The second reason could be that the samples of industrial processing yogurt were taken from the packed product having the chance of contamination during packaging. On the other hand, in the current study, the fresh samples have been taken from the fermentation chamber. While in the case of the local market, the high chances of contamination are inevitable due to improper and substandard storage and handling facilities.

The ratio of *S. thermophilus* to *L. bulgaricus* should be 1:1 for optimum yogurt qualities. In truth, the former is primarily concerned with the generation of acidity, whereas the latter is primarily concerned with the production of flavor-producing components in addition to acidity (volatile fatty acids, acetic acid, acetaldehyde, ethanol etc.). Moreover, to achieve the guaranteed medical benefit to human beings, in fermented milk, the minimum availability of probiotic microbes should be around 9–10 log cfu/mL^51^. The yogurt culture assessment (Table 4) revealed that unbranded (local shops and homemade yogurt) samples, contained both common yogurt species (*S. thermophilus* and *L. bulgaricus*) of lactic acid generating bacteria but their count was found lower than the acceptable range. However, we occasionally see outgrowths of *S. thermophilus* and *L. bulgaricus*, indicating uncontrolled conditions of culture development. Furthermore, the overall count of yogurt culture in branded samples was higher than in unbranded samples and was found in the acceptable range reported by Ouwehand^51^. In contrast, uncontrolled conditions and poor viability might be a reason for lower counts in unbranded yogurt samples. The ratio of *S. thermophilus* and *L. bulgaricus* in yogurt produced by a solar yogurt processing unit was found to be close to 1:1, and the total count of yogurt culture was higher than in unbranded samples due to proper temperature control during fermentation, but less than in branded/company processed samples, as shown in Table 4. These results endorsed the current design.

**Table 4:** Microbiological analysis of the yogurt samples collected from the local market of Faisalabad-Pakistan.

Samples	*S. thermophilus* count (log cfu/mL)	*L. bulgaricus* count (log cfu/mL)	Coliform count (log cfu/mL)
Local shops Yogurt	3.08–4.46	5.73–6.06	1.93–2.00
Homemade Yogurt	3.11–4.48	5.61–6.25	1.34–1.43
Branded/company-processed yogurt	11.00–11.08	10.98–11.04	0.78–1.18
Solar-processed Yogurt	10.18–10.30	10.26–10.34	No-detection

#### Milk adulteration

**Figure 4:**
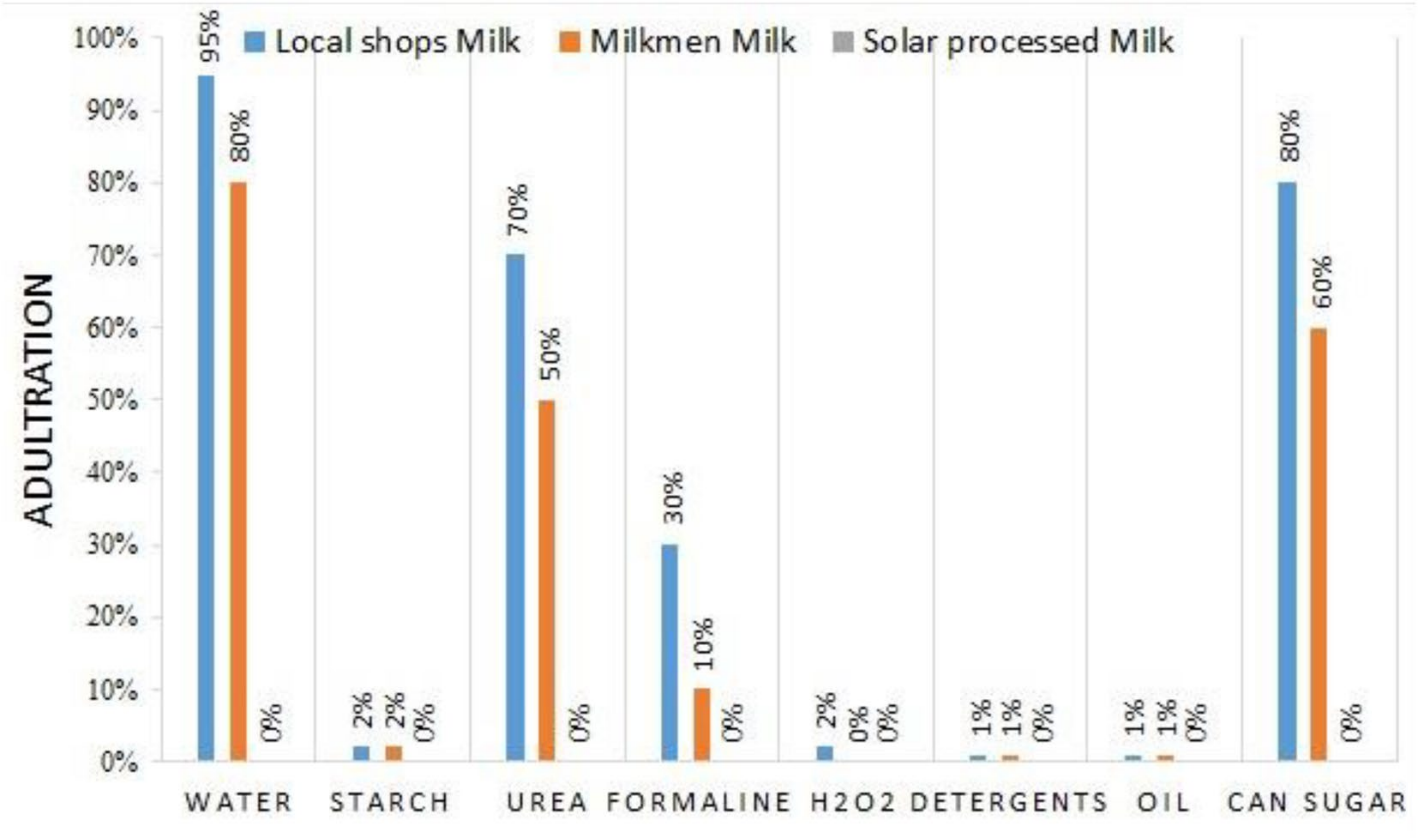
Milk adulteration in local market milk and solar-processed milk.

In comparison to solar-processed milk and yogurt, only local market milk and yogurt samples were evaluated for adulteration. Figure 4 show the results of adulterant detection in local market milk and yogurt, as well as solar-processed milk and yogurt samples. The results showed that local market milk and yogurt samples (Local stores milk/yogurt, and milkmen milk/yogurt) were heavily contaminated with water (70 and 95%), urea (50 and 70%), formalin (10 and 40%), and cane sugar (60 and 80%). Only a minute fraction of starch (2%) in milk and 6% in yogurt samples from local stores, as well as H_2_O_2_ (2%), oil (1%), and detergents (1%) adulteration, were identified in the milk and yogurt samples sold at the local market. Because yogurt was fermented from the same sample milk, the adulteration levels for milk and yogurt from milkmen-supplied milk are the same. All samples of the solar-processed milk and yogurt were confirmed to be free of adulteration.

Adulteration by unscrupulous individuals in the conventional milk supply chain is highly widespread and has been recorded by many authors, therefore the study's findings are found to be consistent with them^11,12,16,23,52^.

### Economic analysis

The economic feasibility of a solar-assisted yogurt processing unit was determined by conducting a comprehensive economic analysis in terms of renewable energy generation from both sources, namely solar thermal using evacuated solar tube collectors for milk heating and PV system for yogurt/milk cooling. The total cost of the Solar aided yogurt processing unit, including all required accessories and installation fees, was 2412 USD. Table 5 lists the data that can be used for economic research.

**Table 5:** Available data for economic analysis.

Unit	Value	Description
Yogurt Processing Unit	900 USD (1 USD = 180 PKR)	That includes Fermentation Chamber (SS-304) Complete Refrigeration System Control Box with Sensor Wiring Thermostat Valve Fabrication and Labor
Solar Thermal Heating System	480 USD	That includes Vacuum Tube Collector Storage Tank Expansion Vessel Pump Control Unit Wiring and Ducting Thermocouples PVC Pipe and its accessories Valves Labor for installation
Solar Photovoltaic System (2kW_p_)	1032 USD	That includes Solar Photovoltaic Modules Tesla (250W Grade A) Hybrid Inverter 2.5 kW Moveable Steel Frame Structure made of GI Batteries 100 Ah AC/ DC Wiring AC/DC Breakers Labor Cost
Total Initial Cost of Complete System	2412 USD	
Expected Life of System	15 years	~ 40,500 h @ 300 sunny days per year and 9 useful hours per day
Salvage value	10%	Of initial cost
Interest rate	7%	Per annum in Pakistan
Insurance and taxes	4%	Of initial cost
Repair & Maintenance	25%	Of initial cost
Total cost of system	4263 USD	
Avg. energy generated by solar thermal system/h	2.0 kWh	With DNI ranging between 700 to 900 Wm^−2^ on a sunny day
Avg. energy generated by PV system/h	0.66 kWh	With DNI ranging between 700 to 900 Wm^−2^ on a sunny day
2.66 kVA generator requires appx	1.2 Lh^−1^	Of gasoline to generate equivalent energy
	0.8 Lh^−1^	Of diesel to generate equivalent energy
	2.66 kWh	Units of electricity per hour
Savings per hour	0.84 USD	Using gasoline @ 0.70 USD/L
	0.58 USD	Using diesel @ 0.73 USD/L
	0.25 USD	Using electricity @ 0.095 USD/kWh

**Figure 5:**
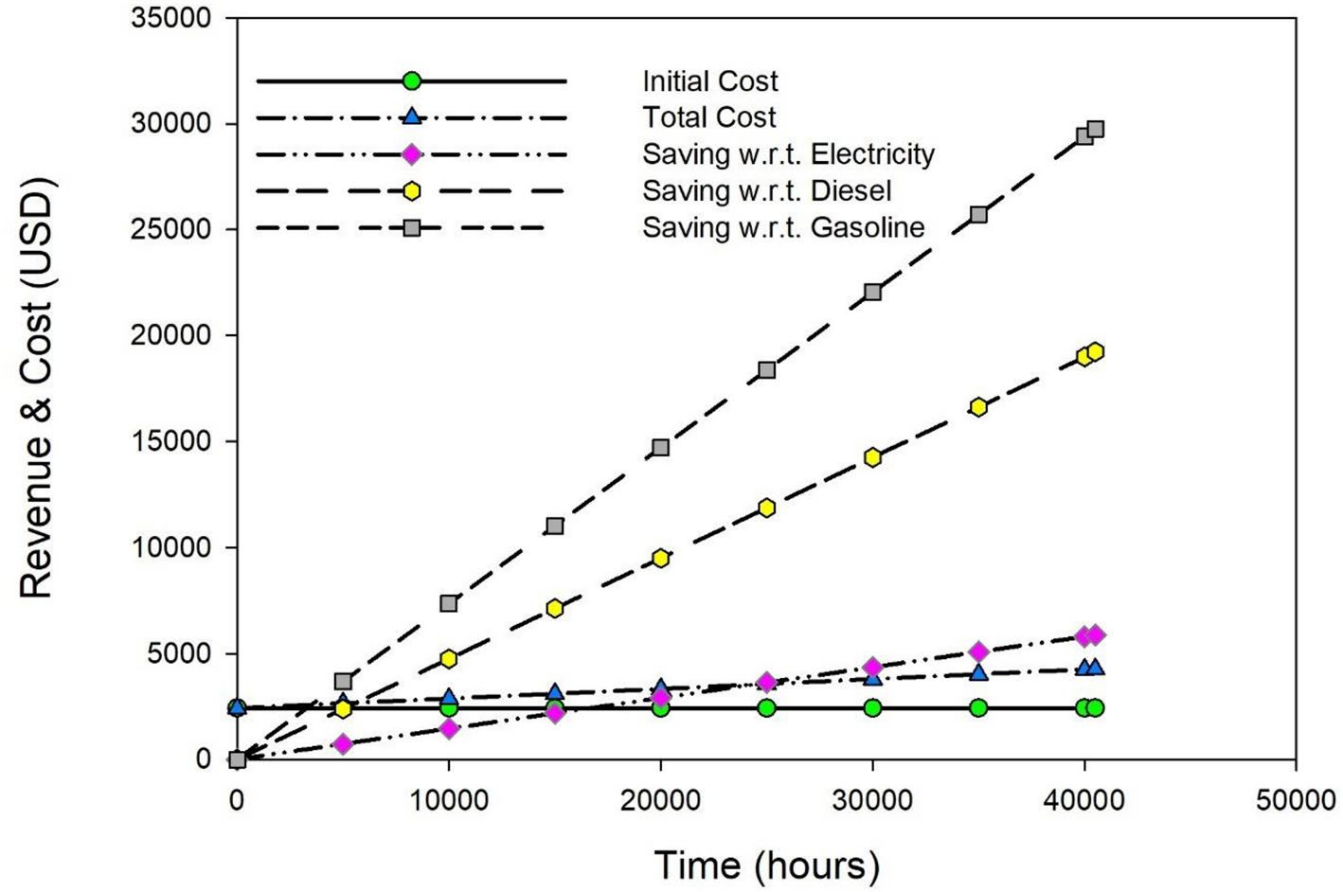
Break-even analysis for the yogurt processing unit in context with fuel saving.

After the initial investment, the individual and overall cost estimation per hour of all economic components and computations revealed that 0.105 USD per hour was necessary to operate the yogurt processing unit. The break-even point analysis was performed based on the available economic data to evaluate the payback duration in relation to other traditional resources. The break-even point for each scenario is calculated by plotting the useful working hours against the expenses, as shown in Fig. 5.

The payback period of the yogurt processing unit was projected to be 3499 (~ 1.3 years), 5619 (~ 2.08 years), and 24,292 (~ 9 years) useful working hours if gasoline, diesel, and electricity were utilized for equal energy generation, respectively as shown in Fig. 5. After the payback period, the total revenue generated over the predicted life expectancy of the yogurt processing unit was estimated to be 27,196, 16,568 and 2,350 USD, respectively, based on the fuel sources of gasoline, diesel, and electricity. The processing cost per liter of milk for yogurt production was calculated to be 0.0189 USD using solar-powered technologies, based on a 50-L batch processed in 9 hours^20,29^, which is significantly less than the estimated processing costs of milk processors, which are 0.2 USD per liter^53^.

#### Payback period based on the processed product

**Figure 6:**
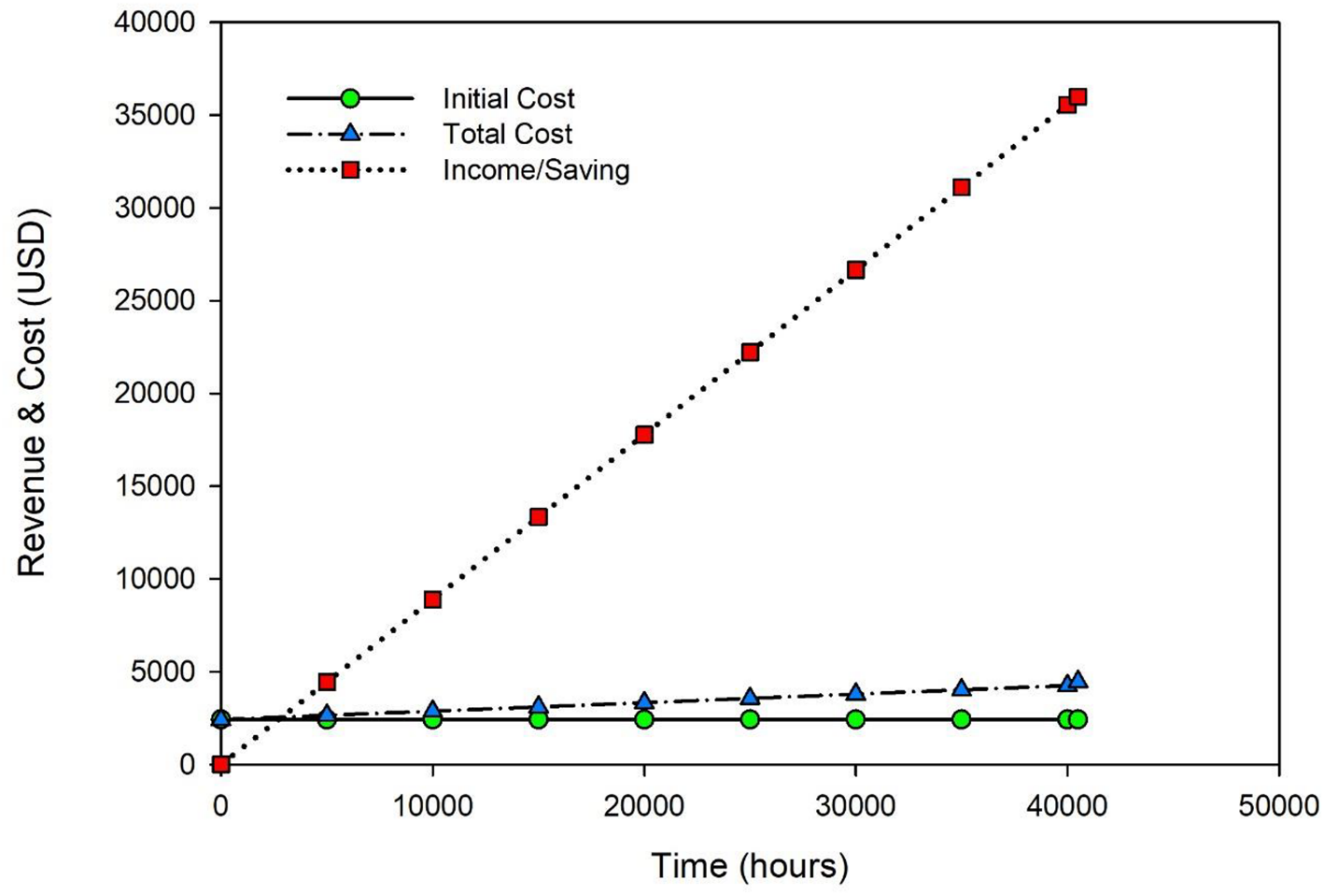
Break-even analysis for the yogurt processing unit in context with product profit.

Lacking the necessary processing and storage facilities, a milkman in rural Pakistan sells milk for USD 0.42 per liter. The milkman can convert milk into a value-added product, stirred yogurt, with the help of a solar-powered yogurt processing plant. In Pakistan, branded packed stirred yogurt costs USD 1.7 per kilogram. If the price of yogurt made with a solar yogurt processing machine is USD 0.7 per kilogram (USD1 per kg cheap form branded yogurt). It is consumer-friendly pricing, and it is simple for milkmen to sell stirred yogurt at this price. The total cost of one kilogram of solar-processed packaged yogurt is estimated by adding the raw milk price of USD 0.42 per kilogram, the processing cost of 0.0189, and the packaging cost of USD 0.1 per kilogram, for a total cost of USD 0.54 per kilogram. So, a rural dairy farmer can save USD 0.16 per kg or USD 8 per day for 50 L of yogurt processing, and the payback period was computed as 533 days (1.78 years) by dividing the entire cost of the system by the savings per day^54^ as shown in Fig. 6.

Basically, the system has been designed to address small and medium-scale milk producers at production sites using solar energy which is not a case of industrial-scale production. The current study justifies the size of the storage tank having 100 L of water capacity to process 50 L of yogurt or milk with 15 tubes of Evacuated Tube Collector (ETC), which is an appropriate size for decentralized application. However, the design can be scaled up by recalculating the size of the storage tank and evacuated tube collector accordingly.

### Carbon emission analysis

**Figure 7:**
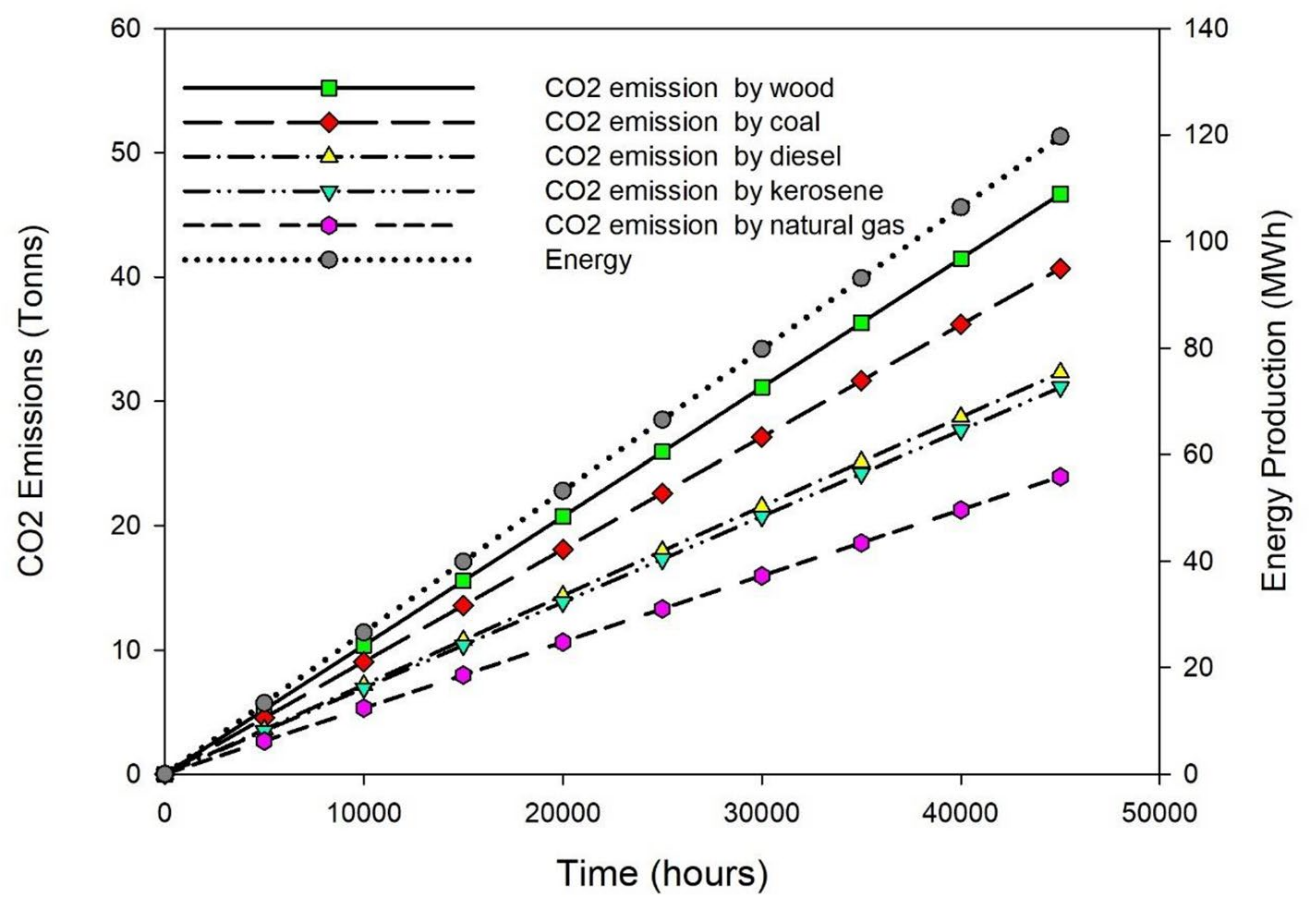
CO_2_ emission savings by solar yogurt processing unit.

The yogurt processing unit was also analyzed for CO_2_ emission savings throughout its estimated life cycle of 40,500 h (15 years) in the context of global warming and climate change. In all seasons, the solar yogurt processing unit can produce roughly 2.66 kW of solar-based energy per hour for on-farm milk/yogurt processing. Based on these findings, it is anticipated that a solar-powered yogurt processing machine can generate about 107.73 MWh of useful process energy throughout the course of its operating life. In Fig. 7, the results of carbon emissions versus energy generation with various non-renewable energy resources are graphically depicted. As shown in Fig. 7, using wood as a fuel source (@ 0.39 kg CO_2_/kWh) will emit 42.015 tons of CO_2_, followed by coal (@ 0.34 kg CO_2_/kWh) 36.63 tons, diesel (@ 0.27 kg CO_2_/kWh) 29.01 tons, kerosene (@ 0.26 kg CO_2_/kWh) 28.01 tons, and natural gas (@ 0.20 kg CO_2_/kWh) 21.55 tons for equivalent energy production for milk/yogurt processing (107.73 MWh). This study found that a solar-assisted yogurt processing unit is a potential green solution for milk/yogurt processing that can successfully solve global warming issues, particularly in terms of carbon emission reduction.

The outcomes show that solar assisted yogurt processing unit gave a quality-oriented product in comparison with locally adopted milk handling and yogurt processing methods. Moreover, the energy required for these processes is also reduced due to solar technology which not only helps to reduce operational cost but also impart a positive impact on climate. The promotion of such novel solar-based dairy processing technologies can help to reduce losses of raw milk in its supply chain and to increase the livelihood of the rural community.

## Conclusions

Milk and yogurt are natural nutritious diets for people of all ages and genders, however, the current study's findings are astounding and contradict this assertion. According to the study's findings, consumers are given a white watery liquid by local milk businesses and milkmen delivering milk to their houses. A considerable number of the samples tested had a foul odor, an odd hue, a thin texture, a nutritious value that had depreciated significantly, and extensive adulteration, particularly by water. It's reasonable to assume that everyone in the milk value chain polluted the milk in some way, either directly or indirectly, but very deliberately. Similarly, the company packed processed milk and yogurt samples that, while found to be free of adulteration, had nutritive values that were trending toward the bottom of the standard ranges because nearly all milk and yogurt processing companies partially skimmed the milk for byproducts before selling it to consumers at a high price.

In comparison to the local market and company-processed milk, raw milk can immediately process using an onsite installed solar-powered yogurt processing unit which showed better results in all quality and consumer acceptance parameters. Because the pure and fresh milk was immediately procured from the UAF Dairy Farm, there was no adulteration and 100 percent hygiene conditions in the solar-processed milk and yogurt. In terms of energy, the break-even point research revealed that a solar yogurt processing unit can pay back in 1.3–9 years, depending on the type of non-renewable source employed for similar energy output. On the basis of product profit, the payback period was projected to be 1.78 years. The cost of processing a liter of milk for manufacturing yogurt was calculated to be 0.0189 USD. The solar-based yogurt processing unit will generate roughly 107.73 MWh of electricity with zero carbon emissions, making it an environmentally beneficial technology. In short, the developed solar yogurt processing unit provides a realistic solution to the local milk value chain's issues. This novel and decentralized solar-based milk and yogurt processing technology allow for on-farm quality milk processing under controlled operating conditions, which can help to alleviate current technology limits for milk farmers as well as quality constraints for customers.

## Data Availability

The raw data supporting the conclusion of this article will be made available by the first and corresponding author, without undue reservation.
